# Mitochondria and Oxidative Stress as a Link between Alzheimer’s Disease and Diabetes Mellitus

**DOI:** 10.3390/ijms241914450

**Published:** 2023-09-22

**Authors:** Ivan M. Veselov, Daria V. Vinogradova, Andrey V. Maltsev, Pavel N. Shevtsov, Elena A. Spirkova, Sergey O. Bachurin, Elena F. Shevtsova

**Affiliations:** Institute of Physiologically Active Compounds at Federal Research Center of Problems of Chemical Physics and Medicinal Chemistry, Russian Academy of Sciences (IPAC RAS), Chernogolovka 142432, Russia; jowent@mail.ru (I.M.V.); maltsevandro@mail.ru (A.V.M.); pnshevtsov@gmail.com (P.N.S.); kustova.ea@mail.ru (E.A.S.); bachurin@ipac.ac.ru (S.O.B.)

**Keywords:** neurodegenerative disease, diabetes, mitochondria, oxidative stress, neuroinflammation, amyloidosis, neuroprotection

## Abstract

This review is devoted to the problems of the common features linking metabolic disorders and type 2 diabetes with the development of Alzheimer’s disease. The pathogenesis of Alzheimer’s disease closely intersects with the mechanisms of type 2 diabetes development, and an important risk factor for both pathologies is aging. Common pathological mechanisms include both factors in the development of oxidative stress, neuroinflammation, insulin resistance, and amyloidosis, as well as impaired mitochondrial dysfunctions and increasing cell death. The currently available drugs for the treatment of type 2 diabetes and Alzheimer’s disease have limited therapeutic efficacy. It is important to note that drugs used to treat Alzheimer’s disease, in particular acetylcholinesterase inhibitors, show a positive therapeutic potential in the treatment of type 2 diabetes, while drugs used in the treatment of type 2 diabetes can also prevent a number of pathologies characteristic for Alzheimer’s disease. A promising direction in the search for a strategy for the treatment of type 2 diabetes and Alzheimer’s disease may be the creation of complex multi-target drugs that have neuroprotective potential and affect specific common targets for type 2 diabetes and Alzheimer’s disease.

## 1. Introduction

Neurodegenerative diseases (NDs) are currently not only a medical problem but also, to a large extent, a social and an economic problem. The main reasons for the lack of significant progress in the development of effective drugs for sporadic forms of NDs’ treatment are a late diagnosis at the stage of an already significant level of neurodegeneration in the brain and the unclear mechanisms of both diseases. Different disease development paths depending on the provoking factors and individual genetic characteristics are suggested for both.

A number of sporadic NDs are strictly age-dependent, and their pathogeneses closely intersect with the mechanisms of aging and pathological conditions that are also age-dependent. Furthermore, patients with metabolic disorders and diabetes have an increased probability of developing age-related NDs, in particular Alzheimer’s disease (AD). The first study to report that T2DM increases the risk of developing AD was the Rotterdam cohort of over 6000 subjects in the Netherlands (1999) [1]. National insurance data from nearly 500,000 men in South Korea showed that T2D was associated with a 60% increase in AD risk (HR = 1.60; 95% CI = 1.29, 1.98) [2]. The symptoms and pathomorphological and biochemical characteristics of these diseases also overlap. Population studies showed that type 2 diabetes (T2D) significantly increases the risk of developing cognitive impairment and dementia, in particular sporadic AD [3]. It is important to understand the mechanisms by which defects in insulin signaling and metabolic disorders can lead to the accelerated progression of AD.

**Figure 1:**
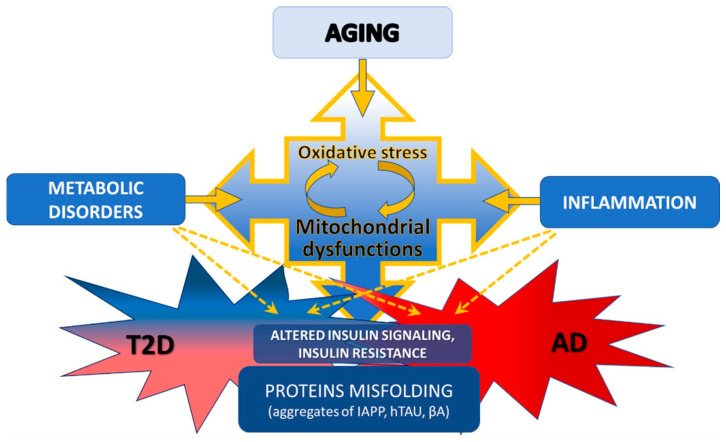
Schematic illustration of the interrelationships of aging, type 2 diabetes (T2D), and Alzheimer’s disease (AD).

Both diseases—T2D and AD—have a complex and unclear etiology in each specific case, but at the same time they have coinciding risk factors. Foremost, there is aging, always accompanied by a vicious cycle of oxidative stress and mitochondrial dysfunction (Figure 1), which, in turn, tightly connected with systemic inflammation and neuroinflammation. Metabolic disorders with a violation of sensitivity to various factors of metabolism regulation, an integral part of which can be considered insulin resistance, are also a characteristic sign not only of T2D and AD but also of aging [4]. Mitochondrial dysfunctions and oxidative stress are significantly much more pronounced compared to physiological aging and are already observed at the prodromal stages of both AD and T2D [4].

This review focuses on the recent findings on the role of mitochondrial impairments and oxidative stress in the development of T2D, T2D-associated neurodegeneration, and AD. Understanding the general mechanisms of the development of diabetes and Alzheimer’s disease is extremely important for the selection of targets when creating drugs for the treatment of both diseases.

## 2. Aging and Insulin Resistance

The mitochondrial theory of aging and the pathogenesis of AD is based on the idea of a vicious cycle in which the accumulation of mtDNA mutations with age leads to respiratory chain dysfunction, increasing the production of oxygen radicals, which leads to the further accumulation of mtDNA mutations. Finally, in the case of a genetic predisposition and in the presence of exogenous factors, a bioenergetic crisis leads to the increasing dysfunction and degeneration of organs and tissues [5].

The insulin signaling pathway is one of the most conserved evolutionary pathways that control aging [6]. Although, in most vertebrates, the hormonal role of the glucose level regulator belongs to insulin synthesized by the pancreas, the ability to synthesize and secrete insulins, as well as the insulin signaling pathway, is present in different cells and tissues [7]. Accordingly, it can be assumed that disturbances in the insulin signaling pathway, in particular insulin resistance, may be present in various organs and cells and determine a similar pathology of metabolic regulation in various diseases [8]. However, insulin resistance is not a systemic disorder in all cases of metabolic disorders. The presence of peripheral insulin resistance does not necessarily mean the presence of insulin resistance in the brain, and vice versa: insulin resistance in the brain is not always accompanied by peripheral insulin resistance [9]. For T2D, central insulin resistance is characterized by a decrease in insulin receptor expression, insulin binding to receptors, and abnormal insulin signaling. The same features of insulin resistance are also inherent in brain cells in AD [10]. 

There is significant evidence that insulin resistance and central insulin resistance in particular are strongly associated with the impairments of mitochondrial homeostasis and oxidative stress. The presence of markers of mitochondrial dysfunctions, such as decreased mtDNA, mRNA levels for mitochondrial genes, and protein expression of respiratory chain subunits, reduced the size and density of mitochondria and reduced the activities of respiratory chain complexes shown in various insulin-resistant states, including obesity, aging, T2D, and others. Moreover, a high-fat diet simultaneously induces insulin resistance and the appearance of the above-mentioned mitochondrial dysfunctions, including a decrease in the expression of PGC1a and PGC-1b, which regulate mitochondrial biogenesis [11]. 

In the pancreas, mitochondria often colocalize with the secretory insulin granules that may facilitate metabolism–secretion coupling. Insulin secretion is realized through the ATP-dependent closure of ATP-sensitive K^+^ channels and the subsequent opening of voltage-dependent Ca^2+^ channels on the plasma membranes of β-cells. ATP is generated by oxidative phosphorylation (OXPHOS). The increased intracellular concentration of Ca^2+^ ultimately initiates insulin exocytosis, in a process known as glucose-stimulated insulin secretion. Mitochondrial OXPHOS in islet β-cells is crucial for glucose-stimulated insulin secretion, and the impairment of mitochondrial functions inevitably leads to a disruption of insulin secretion [12].

For a long time, it was believed that the brain is an insulin-insensitive organ. However, recently it was shown that insulin penetrates into the brain, and its receptors are expressed in all types of brain cells. Insulin plays a multi-functional and sometimes ambivalent role in the regulation of the functions of various brain cells. It regulates not only food-related, glucose metabolism in the brain but also other, in particular cognitive, functions of the CNS. Insulin may regulate systemic functions such as hepatic glucose production, lipolysis, lipogenesis, inflammation, fertility, and the sympathoadrenal response to hypoglycemia. Deficiency in the action of insulin in the brain can be caused either by low levels of insulin in the brain or by a deficiency or an impairment of the insulin receptors in brain cells. Since insulin receptors in the brain are not involved in the classic negative feedback between glucose and insulin levels, blood insulin levels are largely independent of insufficient brain insulin activity, making it difficult to test for brain insulin resistance in living subjects. Reduced brain insulin action (or brain insulin resistance) can be observed in obesity, T2D, aging, and AD, suggesting a possible link between metabolic and cognitive health [13].

The neurons and β-cells of the pancreas have a significant similarity: they are electrically excitable cells with similar ion channels (K^+^-ATP channel and voltage-dependent L-type Ca^2+^-channel), mechanisms of responses to the depolarization of cell membranes, and implementations of basic physiological functions through exocytosis [14]. Common signaling mechanisms are a response to the similar physiological stimuli in the pancreas and brain, and similar signaling disorders may be the underlying cause of the common pathology [15].

## 3. T2D and AD as Amyloidoses

Animal studies confirmed that modeling a T2D-specific pathology can provoke or exacerbate the development of the characteristic features of AD, including the development of amyloid plaques, abnormal neurite curvature, and neurodegeneration [16]. Thus, for transgenic mice with cerebral amyloidosis (AD model-5xFAD model), after the induction of experimental diabetes with streptozotocin (STZ), a significant decrease in the level of insulin in the brain was observed without changes in the expression of insulin receptors or, simultaneously, an increase in the level of the amyloid precursor protein (APP), beta-secretase activity, and, accordingly, the concentration of β-amyloid peptides (βA) 1-40 and 1-42, as well as C-end fragment C99 [17], which indicates both the appearance of insulin regulation impairment and the stimulation of the development of cerebral amyloidosis.

T2D, like AD, belongs to the so-called amyloidoses, i.e., diseases characterized by insoluble amyloid-like inclusions. For AD, the main component of amyloid plaques is a specific βA, which is formed as a result of the targeted proteolysis of the APP, and, for diabetes, it is amylin (or islet amyloid polypeptide; IAPP), as well as insulin. Insulin is found in the pancreas as an inactive zinc hexamer; when released into the blood serum, the hexamer dissociates into a dimer and then into a monomer, which is a physiologically active form of insulin [18]. The insulin monomer is prone to aggregation with the formation of amyloid aggregates; amyloid-like fibrils can form even at the injection site of insulin in patients with insulin-dependent diabetes. IAPP, which is, in a sense, an insulin antagonist, has a much greater tendency to form amyloid, and it is the main component of amyloid deposits in the intercellular space and in the β-cells of the pancreas islets of Langerhans. It is important to note that, unlike human and cat IAPP, rodent IAPP does not have amyloidogenic properties [19]. Amyloid formation in the islets of Langerhans is one of the main causes of the death of hormone-producing pancreatic cells in both type 1 diabetes and T2D, when insulin resistance leads to a compensatory increase in insulin and IAPP production, which, in turn, leads to an increase in amyloidosis in the islets of Langerhans and their involution.

It is with proteins prone to the formation of amyloid aggregates that the violation of mitochondrial functions can be associated both with AD or Parkinson’s disease and diabetes. It was shown that protein aggregates of βA, the hyperphosphorylated tau protein (pTau), and alpha-synuclein (αSyn) co-localize and directly interact with individual elements (CypD, VDAC, ANT, and ATP synthase) of the complex polyprotein complex of the mitochondrial permeability transition pore (mPTP) [20] and not only increase the probability of mitochondrial permeability transition but also induce the opening of mPTP [21]. This may contribute to the accumulation of mitochondria prone to opening mPTP and triggering cell death cascades. The characteristic amyloid-forming protein in T2D is the hormone IAPP, the monomeric form of which is co-secreted with insulin and modulates satiety signaling in the brain. Insulin resistance leads to a compensatory increase in the synthesis and secretion of insulin by the beta cells of the islets of Langerhans and, accordingly, IAPP. This is accompanied by the induction of endoplasmic reticulum stress and the impaired folding of the amyloid-producing protein, IAPP. The oligomeric form of IAPP deregulates calcium homeostasis and is able to form ion-permeable channels in membranes, including mitochondrial ones, which leads to a decrease in the survival of pancreatic β-cells [22,23]. At the same time, oligomeric forms of this protein can also be formed in serum and disrupt the functioning of the vascular pericytes of the blood–brain barrier and its permeability [24]. Extracellular fibrillar aggregates of IAPP were found both in the brains of patients with T2D and in the brains of patients with AD [25,26]. In addition, the formation of IAPP aggregates was observed, including IAPP adducts with one of the main products of lipid peroxidation (LP), 4-hydroxynonenal (4-HNE), both inside neurons [27] and inside microglial cells [28]. It is important to note that the colocalization of pTau and IAPP was found in the neurofibrillary tangles in the neurons of AD patients [28]. Since a direct role in inducing the opening of mPTP is shown for βA [21], we can assume such activity for IAPP oligomers. In experimental models of diabetes, the sensitivity of brain mitochondria to the opening of mPTP is increased in most cases. In the brain of diabetic Goto-Kakizaki (GK) rats, the mPTP opening threshold and the calcium capacity of brain mitochondria are lower than in control rats [29,30].

## 4. Mitochondrial Dysfunction in T2D and AD

Mitochondrial dysfunction is one of the key links in the pathological processes of both NDs and diabetes. The causes of mitochondrial dysfunction may be a violation of the systemic functions in the cell or the direct effect of the effectors on the organelles. The characteristics of mitochondrial dysfunction are changes in the number of mitochondria in the tissues, deep ultrastructural abnormalities of the organelles, impaired mitochondrial biogenesis, the decreased activity of the mitochondrial multienzyme complexes, impaired ATP synthesis, impaired calcium homeostasis, the decreased threshold of the mPTP opening, and the excessive formation of reactive oxygen species (ROS); a detailed review of T2D-associated mitochondrial dysfunction can be found in [29].

### 4.1. Mitochondria Quality and Quantity

Mitochondrial impairment in T2D and AD is facilitated by the disruption of mitochondrial autophagy and biogenesis [31]. Mitochondrial biogenesis is mainly controlled by the PPAR/PGC-1α system. PGC-1α is a transcription coactivator that interacts with a wide range of transcription factors and regulates the expression of key genes involved in mitochondrial biogenesis, adaptive thermogenesis, and metabolism. In addition, PGC-1α acts as a coactivator for PPARα and δ, which, in turn, regulate the expression of genes involved in mitochondrial fat catabolism [32].

Numerous studies showed that in diabetes there is a decrease in the expression of PGC-1α itself, as well as a decrease in the expression of genes sensitive to PGC-1α and nuclear respiratory factor-1 (NRF-1), which encode oxidative enzymes [33,34,35]. The use of antidiabetic drugs (metformin, thiazolidinediones, and empagliflozin), as well as regular physical activity, restores the level of PGC-1α in diabetic in vivo models [29].

A decrease in the level of PGC-1α was also found in the hippocampus of patients with AD post mortem and in the brain of 3xTgAD mice [36,37]. A direct correlation between the density of neuritic plaques and the level of PGC-1α and an inverse correlation between the content of βA and PGC 1α were shown. A correlation was also found between the levels of PGC-1α expression and amyloidogenesis [35]. Experiments in vitro showed βA, in an oligomeric form, caused a decrease in the amount of PGC-1α and SIRT1 and a disruption of the PGC-1α/SIRT1 interaction. Under the influence of soluble βA oligomers, the pathological redistribution of PGC-1α from the nucleus to the cytosol occurs [38].

Changes in the expression level of PGC-1α affect the number and density of mitochondria in different types of neurons [39] and modulate the processes of mitochondrial fusion and fission [40]. An increase in PGC-1α gene expression with a corresponding normalization of the protein level leads to the restoration of the energy functions and insulin sensitivity in cells [33]. However, PGC-1α overexpression has a negative effect on heart [41], muscle [42], and brain [43] cells. To avoid the side effects of PGC-1α overexpression, various approaches are being considered for the mild indirect regulation of PGC-1 activity through an increase in adenosine monophosphate-activated protein kinase (AMPK) activity and/or the inhibition of SIRT, as well as by influencing the PPAR family of receptors, the main transcription factors regulated by PGC-1α [44].

PPARγ is abundant in the brain and is vital for learning processes [45]. PPARα regulates mitochondrial metabolism (including the fatty acid β-oxidation pathway), energy processes, glucose metabolism, the redox state, and glutamatergic, cholinergic/dopaminergic neurotransmission. The activation of the receptor leads to the metabolic coupling of neurons and astrocytes, promotes the formation of dendrites, and prevents the disruption of synaptic transmission [45].

At the same time, PPARγ plays an important role in the prevention/development of pathologies such as obesity, diabetes, and neuroinflammation [46]. In addition, PPAR-α is involved in the metabolism of the beta-amyloid precursor protein (APP) in the brain, and directly or indirectly through Aβ can also influence tau protein phosphorylation [47]. PPARγ and PPARα agonists have an antidiabetic effect and a wide range of activities aimed at counteracting many elements of AD pathology [48].

The levels of the gene and protein expression of PPARγ, AMPK, and insulin-degrading enzyme (IDE) significantly decrease after the STZ induction of T2D in mice with an AD model (APPSwe/PS1), while in transgenic APPSwe/PS1 mice, as well as in wild-type mice with an STZ T2D model, there were no significant differences from control animals. The IDE metalloprotease not only plays a key role in the degradation of insulin but also is involved in the degradation of monomeric forms of βA. The PPARγ activator rosiglitazone, as well as the AMPK activator AICAR, leads to an increase in the level of IDE expression and, accordingly, to a decrease in the level of βA(1-40) and βA(1-42) as well as an alleviation of cognitive impairment [49].

### 4.2. Energy Metabolism in Mitochondria

A disturbance of the energy metabolism of mitochondria leads to the accelerated aging of cells and the body as a whole. In experiments with 3xTg AD mice, the expression of complex IV of the mitochondrial respiratory chain (MTCO1 subunit) and the activity of complex II + III were found to decrease with the progression of the disease [37]. Previously, it was shown that in the blood of AD patients there is already a change in the level of expression of the genes of the individual complexes of the respiratory chain of mitochondria at the early stages of the disease. It was shown that complex IV is the most vulnerable in AD: there is a decrease in the number of individual subunits of the complex and, as a result, its overall activity [50]. Changes in the expression of genes encoding proteins of oxidative phosphorylation lead to a decrease in the efficiency of the mitochondrial electron transport chain and, as a result, to the formation of an excess amount of ROS [50].

In the brain of diabetic rats of the Goto-Kakizaki (GK) line, the formation of ATP is reduced. With age, the efficiency of oxidative phosphorylation in the brain of these rats decreases to a greater extent compared to normal rats [30]. Also, for this line, a decrease in the activity of complexes I, II/III, and IV of the respiratory chain of liver and kidney mitochondria was experimentally shown [51]. Regular aerobic physical activity improves energy metabolism, glutamate dehydrogenase activity, and expression of ROS-sensitive mitochondrial marker aconitase in the liver and kidney of diabetic animals. But, in the muscle skeletal tissue of GK rats, it improves the enzymatic activity of complexes I, II, and III. Complex IV of the mitochondrial respiratory chain does not differ, i.e., mitochondrial functions in muscles do not depend on insulin resistance [52].

In another in vivo diabetic model, Zucker rats (ZDF), a significant decrease in the activity of complexes I, II/III, and IV, as well as in the level of ATP in brain and liver mitochondria, was shown [53]. At the same time, the opposite results were obtained for liver and brain tissues regarding changes in ROS homeostasis. In the brain of ZDF rats, the level of oxidative and nitrosative stress was higher compared to control rats, while a decrease in ROS production was recorded in the liver. Also, this line of rats with a model of diabetes is characterized by an increase in the level of protein acetylation in the mitochondria of the kidneys of ZDF rats, which is associated with a decrease in the activity of mitochondrial deacetylase sirtuin 3 (SIRT3). As a result, the activity of the isocitrate dehydrogenase and superoxide dismutase SOD2 of mitochondria also decreases in the kidneys of ZDF, and the expression of NAD-degrading enzyme CD38 increases. Changes in the activity of key enzymes of the mitochondrial antioxidant system and the NAD^+^/NADH ratio provoke an increase in mitochondrial oxidative stress [54].

AMPK is serine/threonine protein kinase that acts as an energy sensor in cells and plays a key role in the upregulation of catabolism and the inactivation of anabolism. AMPK also partly controls antioxidant defense and insulin signaling [55]. The regulation of this kinase is impaired in diabetes, obesity, and NDs [56]. In AD, the simultaneous phosphorylation of AMPK and mTOR, a factor responsible for the regulation of autophagy [57], is observed, mainly in the localization of phosphorylated tau. The overactivation of mTOR is observed in patients with AD [58] and leads to the accumulation of oxidative proteins impairments in the brain [59]. mTOR overactivation is also responsible for the inhibition of IRS1 and plays a role in the appearance of insulin resistance in AD [60]. In addition, in cells with excessive phosphorylation of AMPK and mTOR, the content of mitochondrial antioxidant enzymes is reduced, and, accordingly, the level of mtDNA and protein oxidation is increased [61].

### 4.3. Calcium Homeostasis

Ca^2+^ ion transport is essential for mitochondrial function and cellular metabolism. The dysregulation of Ca^2+^ metabolism in mitochondria is involved in the pathogenesis of several diseases such as insulin resistance, T2D, diabetes-related cardiac disease, heart failure, ischemia, reperfusion injury, brain aging, neurodegenerative diseases, and cancer [62].

The involvement and significance of the main cellular functions of mitochondria in Ca^2+^ signaling varies depending on the cell type [63]. Insulin secretion in pancreatic cells is regulated by mitochondrial voltage-dependent Ca^2+^ channels on the plasma membranes of β-cells [12]. Recent studies confirmed the important role of mitochondria in Ca^2+^ regulation in brain; in particular in synaptic neurotransmitter vesicle release and in dendrites, mitochondrial Ca^2+^ regulation affects synaptic plasticity [64].

The uptake of Ca^2+^ ions into the mitochondria intermembrane space occurs through VDAC in the outer mitochondrial membrane. It is a multifunctional protein permeable for small molecular metabolites. VDAC controls the energetic and metabolic crosstalk between the cytoplasm and the mitochondrial matrix. It regulates a number of processes crucial for normal cell physiology, including Ca^2+^ homeostasis, ATP production, and mitochondrial mediated apoptosis [65]. VDAC overexpression was shown to induce apoptotic cell death and to be common in many diseases (T2DM, cancer, Alzheimer’s disease, Parkinson’s disease, and cardiovascular diseases). VDAC dysregulation may be a common mechanism in the development of T2D and NDs [66,67]. However, it is unclear whether the diseases cause VDAC overexpression or VDAC overexpression causes these pathologies.

The Ca^2+^ concentration balance between the cytoplasm and mitochondrial matrix is regulated by a multicomponent MCU complex assembled from a number of pore forming subunits including MCU, MCU paralog—MCUb, the essential MCU regulator—EMRE and of regulatory subunits including mitochondrial calcium uptake 1 (MICU1), MICU2, MICU3 and MCU regulator 1 (MCUR1). Currently, three different mechanisms of Ca^2+^ release are described via the Na^+^/Ca^2+^ exchanger (mtNCX), the H^+^/Ca^2+^ exchanger (mtHCX), and the mitochondrial permeability transition pore (mPTP). The development of diabetes leads to an increased Ca^2+^ uptake in several in vivo and in vitro models [68]. The data on the role of MCUb in type 2 diabetic models are controversial, whereas MCUb in the brain is supposed to play neuroprotective roles for strokes and NDs [64].

There is also evidence of NCLX expression increase caused by diabetes [69]. 

Mitochondrial Ca^2+^ overload is a common neurotoxic mechanism implicated in various NDs [63]. The loss of NCLX expression and a reduction in the mitochondrial calcium uniporter channel (mtCU)-associated proteins, MICU1 and MCUB, correlates with AD progression in sporadic AD patients and 3xTg-AD mice [68]. The loss of neuronal NCLX in 3xTg-AD mice sensitizes mitochondria to the mPTP opening. In another study using NCLX-KO mice and neurons, it was demonstrated that the genetic loss of function of NCLX is linked to intellectual disability [70].

The cyclosporine A-sensitive mPT pore is considered to act as a system for Ca^2+^ release. Its structure is not clearly known, yet it is proven to be involved in the development of both NDs and T2D [71].

## 5. Oxidative Stress as a Factor in the Development of Pathology in AD and T2D

Oxidative stress plays an important role in the development of pathological disorders in both AD and T2D; it is associated with an increase in the formation of ROS and the accumulation of oxidative damage to macromolecules [72,73,74,75,76,77,78]. Normally, ROS production has a physiological regulatory role. At the periphery, ROS facilitate insulin signaling in response to insulin itself, in particular, by inhibiting protein phosphatases [79]. In the brain, ROS can be involved in long-term potentiation, synaptic signaling, and even in memory mechanisms [80]. However, when certain thresholds of these influences on metabolism and nervous processes are exceeded, ROS already have undesirable effects. It was shown that βA and human IAPP, in contrast to rodent IAPP, increase the levels of ROS formation in the cytoplasm and mitochondria [81], which causes a significantly higher level of oxidative damage to proteins, nucleic acids, and lipids compared to normal aging.

In T2D, persistent hyperglycemia leads to intense production of ROS [82], in particular in mitochondria [83]. Disturbances in the functioning of antioxidant defense systems were also found [84]. Mitochondrial dysfunction and oxidative stress are key points in the pathogenesis of AD and T2D [85]. Impairment of the functioning of the respiratory chain of mitochondria in the brain, leading to oxidative stress, were also found in the STZ model of T2D in rats [86]. At the same time, in rats with the STZ T2D model, the content of lipid peroxidation products in the hippocampus was increased, and the SOD content was markedly reduced. Also, the amount of mtDNA and the number of proteins of the mitochondrial respiratory chain are reduced [87].

Insulin signaling plays a protective role and regulates mitochondrial function. It was established that intense insulin signaling protects neuroblastoma cells from βA-mediated oxidative stress through the activation of the Akt pathway, inhibition of proapoptotic factors, and maintenance of the mitochondrial potential [88]. The appearance of insulin resistance and neuropathy in diabetes is also closely associated with oxidative stress [89]. At the same time, insulin resistance provides an increase in the mitochondrial level of ROS and, as a result, leads to neuronal apoptosis [90]. Impaired insulin signaling leads to mitochondrial dysfunction, oxidative stress, and the accumulation of advanced glycation end products (AGEs). The inhibition of insulin receptors by βA oligomers causes oxidative stress and impaired insulin signaling in the hippocampus [91]. Also, in the culture of neuroblastoma and hippocampal cells, the direct binding of βA to one of the most important antioxidant enzymes, catalase, and the inhibition of its activity was shown, resulting in the accumulation of hydrogen peroxide [92]. Moreover, a hypothesis about the development of oxidative stress specifically mediated by βA-catalase interaction was proposed [93]. It is important to note that a significant positive correlation was found between the development of T2D and the presence of rare hereditary diseases associated with mutations in the catalase gene—acatalazemia and hypocatalazemia [94]. One of the unique features of pancreas β-cells is the low expression of catalase and other antioxidant enzymes, along with a significant content of mitochondria, which, accordingly, leads to their increased vulnerability to oxidative stress [95]. Thus, the accumulation of βA oligomers in the brain and/or pancreatic gland can lead to neuronal and cellular dysfunction in AD and T2D [91].

Nevertheless, an increase in the amount of ROS in diabetics is mainly associated with the increased formation of superoxide in mitochondria and the protein kinase-C-dependent overactivation of NADPH oxidase. An increase in the expression of mRNA in the gp91phox and p22phox subunits of NADPH oxidase and a corresponding increase in lipid peroxidation products were found in the brains of T2D patients and in the brains of mice with diabetes models [96]. In mice with the alloxan model of diabetes, an increased level of superoxide, protein oxidation products, and lipid peroxidation products were found in the brain, often in combination with a decrease in the activity of SOD, glutathione peroxidase, and catalase [97]. In the brain of Zucker rats with a model of diabetes, a decrease in ATP synthesis and an increase in the level of ROS and, accordingly, lipid and protein oxidation products were found [54]. A high-fat diet also leads to oxidative stress, mitochondrial dysfunction, the activation of pro-inflammatory factors in the hippocampus, and significant cognitive decline [98].

One of the important factors of the regulation of oxidative stress and the mitochondrial functions associated with the induction of apoptosis and the regulation of the mPTP is the adapter protein from the *shc* family—P66shc. This protein is a functional regulator of the mammalian lifespan and is expressed in many cells, including neurons and microglial cells, where its overexpression reduces the expression of glycolysis proteins and increases the activity of the mitochondrial electron transport chain, provoking ROS production and increasing vulnerability to the toxic effect of βA [99]. The P66Shc protein is also expressed in pancreatic β-cells, and its overexpression mediates the appearance of insulin resistance and the impairment of secretory function [100]. It is assumed that P66Shc can stimulate oxidative stress in three possible ways: by stimulating the assembly and increasing the activity of membrane NADPH oxidases, by decreasing the expression of antioxidant enzymes, and by provoking ROS production by mitochondria, which may be associated with the induction of the mitochondrial permeability transition [101]. Expression of the p66Shc gene is significantly increased in T2D, and knockout of this gene is able to prevent cognitive impairment in mice with STZ-induced T1D and T2D models; increase resistance to factors associated with aging—oxidative stress, hyperglycemia, hypercholesterolemia, and ischemia [101,102]; prevent endothelial impairment in hyperglycemia; and reduce cognitive deficits in transgenic mice PSAPP with a model of Alzheimer’s disease (cerebral amyloidosis model) [103,104].

As mentioned above, in chronic glucose metabolism disorders (for example, in T2D), hyperglycemia leads to the formation and accumulation of the non-enzymatic glycation end products of proteins, nucleic acids, and lipids—advanced glycation end products (AGEs). This process is one of the fundamental causes of aging, a mechanism for the development of oxidative stress, and a characteristic feature of T2D. During glycation, numerous prooxidant molecules are synthesized, including reactive glyoxal aldehydes, methylglyoxal, and 3-deoxyglucoson. Many glycation products, such as Amadori products, subsequently react with oxygen to form significant amounts of ROS. The interaction of AGEs with a specific AGEs receptor (RAGE) enhances oxidative stress, decreasing the activity of antioxidant enzymes and the level of glutathione and increasing the production of ROS, in particular, due to the activation of mitochondrial NADPH oxidases [105]. In contrast, glycation may be a factor predisposed to the formation of toxic forms of βA [106]. It was shown that glycation mechanistically ensures the refolding of globular albumin from a predominantly α-helical structure to a cross-β structure common for amyloids, which is usually common for all amyloids [107]. The glycation of the amyloid-forming protein IAPP characteristic of T2D also significantly increases its aggregation with the formation of Congo Red positive aggregates [108].

It is important to note that the degree of glycation of the βA correlates not only with its aggregation and the formation of extracellular senile plaques but also with the hyperphosphorylation of the tau protein and the corresponding formation of intracellular neurofibrillary tangles, which, like senile plaques, are pathomorphological markers of AD. In insulin-producing beta cells of the pancreas, the tau protein is also actively expressed, which plays a significant role in the production and secretion of insulin [109]; its knockout provokes the development of the symptoms of diabetes [110], and the presence of a hyperphosphorylated form of the tau protein was shown in the beta cells of patients with T2D [111]. In contrast, the presence of IAPP in the brain of AD patients and its colocalization with the hyperphosphorylated tau protein was shown [28]. The tau protein can also undergo glycation, which leads to the impossibility of its proteolysis and to the further provocation of the formation of free radicals [112,113]. 

It was shown that in the brain of AD patients, an increased content of heme oxygenase-1 (HO-1) colocalizes with tau-containing neurofibrillary tangles [114]. Significant expression of HO-1 was found in the hippocampus, cortex, and subcortical white matter of the brain in AD, and immunoreactivity for HO-1 also coincides not only with neurofibrillary tangles but also with senile plaques [115]. HO-1 breaks down prooxidant heme to biliverdin, which is then reduced to bilirubin, releasing CO and ferrous iron. HO-1 expression is upregulated under oxidative stress, which can lead to a reduction in both oxidative stress, due to the radical scavenging activities of bilirubin, and neuroprotection, due to the anti-apoptotic and anti-inflammatory activity of carbon monoxide. In rats with a high-fat T2D model, elevated HO-1 activates AMPK and improves insulin sensitivity [116]. In rats with STZ-induced diabetes, reduced levels of the mitochondrial ADP/ATP transporter and cytochrome C oxidase are restored to the level of control animals upon induction of HO-1 [117]. The protective role of HO-1 against oxidative stress and metabolic dysfunction makes it a very interesting therapeutic target for both diabetes and AD drugs’ development. At the same time, a correlation was shown between the level of HO-1 protein and a cytoprotective or cytotoxic effect, which indicates the presence of a threshold for the upregulation of HO-1, above which the formation of the byproduct of this enzyme—free iron—becomes toxic. In addition, the available data suggest that HO-1 activation associated with Nrf2 and the corresponding expression of antioxidants and some other genes lead to the protection of neurons and glial cells, and Nrf2-independent HO-1 activation has a neurotoxic effect [118]. 

## 6. Relationships among Insulin Resistance, Inflammation and Mitochondrial Dysfunction

The data from numerous studies indicated that the process of inflammation is characteristic for NDs, including AD, as well as diabetes [119]. Moreover, neuroinflammation was implicated in the etiology of AD, but its contribution to disease progression has not yet been sufficiently studied [120]. Astrocytes and microglial cells are the main cell types involved in the inflammatory responses in the central nervous system (CNS). Several studies described that βA, pathogenic infection, or cellular debris induce an initial inflammatory stimulus that activates microglia, allowing neuronal plasticity and synaptic connectivity to be maintained [121,122]. It was shown that microglia internalize and destroy βA aggregates, but, as the pathological process develops, microglial cells acquire a “toxic” phenotype due to chronic activation and continue to produce pro-inflammatory mediators [123]. In animal models and in human brain tissue, both amyloid plaques and neurofibrillary tangles colocalize with activated glial cells. Various studies reported pathological astrogliosis, both in AD patients and in transgenic animals, characterized by an increase in the glial fibrillary acidic protein (GFAP) and a distinct cellular hypertrophy, which somehow correlates with the severity of cognitive impairment in AD patients [124].

According to the latest ideas, the molecular mechanisms of the development of T2D are, in many respects, similar to the mechanisms of the development of neurodegeneration, and the development of T2D is also accompanied by inflammation. Some authors even call AD type 3 diabetes. Insulin resistance, insulin deficiency, and elevated glucose levels are characteristic of the early stages of AD development, and patients with T2D diabetes are 1.4–2 times more likely to develop AD [125].

AD and T2D are characterized by changes in the levels of key inflammation markers such as the c-reactive protein, tumor necrosis factor α, interleukin-6, interleukin-1, etc. [126]. In NDs, these factors are secreted by microglial cells, which are phagocytic immune cells that perform the function of utilizing dead neurons. Microglia tend to be activated in the directions of both pro-inflammatory and anti-inflammatory reactions [127].

As T2D develops, hyperexpression of interleukin-1β is observed in pancreatic β-cells, which indicates inflammation, the process of apoptosis of these cells, and, as a result, impaired insulin secretion [128]. Pro-inflammatory cytokines in T2D can easily penetrate a blood–brain barrier damaged due to pathology, cause a neuroinflammation reaction, and induce the production of inflammatory cytokines in the brain, which leads to the development of dementia [129].

Inflammation results in mitochondrial dysfunction, while the converse, that mitochondrial dysfunction induces inflammation, is also true [130,131,132]. Inflammatory signals initiate in response to a pathogen or a “foreign” agent. Novel data now suggest mitochondria and/or mitochondrial components could mimic a pathogen—sending a “danger” signal and triggering an inflammatory mitochondrial dysfunction also lead to the release of mitochondrial components into various cellular compartments and into the intercellular space, causing an inflammatory response. One of the basic mitochondrial agents to activate the inflammatory response are the so-called mitochondrial damage-associated molecular patterns (mtDAMPs). According to the endosymbiotic theory of origin, mitochondria are of bacterial nature, and, at the early stages of evolution, they entered eukaryotic cells from the outside. mtDAMPs are macromolecules that, when formed intracellularly or extracellularly, can cause a strong inflammatory response due to the fact that their evolutionary nature is bacterial [133]. Mitochondrial pro-inflammatory molecules include, but are not limited to, mitochondrial DNA (mtDNA), adenosine triphosphate (ATP)—only when extracellularly released, the main inner membrane’s phospholipid cardiolipin, mitochondrial transcription factor A (TFAM), cytochrome c, formyl peptides, and RNA. The immunogenic properties of these molecules were previously reviewed [131]. For example, mtDNA contains unmethylated CpG motifs similar to bacterial ones. During normal cell functioning, mitochondria and its mtDNA are utilized through mitophagy, but, in case of disturbances, such mtDNA fragments are released and can activate macrophage inflammasomes, which induces the production of interferon I and interleukin-1β, thereby causing an inflammatory response [134,135]. The most studied way of the mtDNA-dependent induction of inflammation is the activation of the Toll-like receptor-9 binding pathway, which triggers microglial inflammatory responses [136,137]. Damaged mitochondria generate ROS, which, in turn, activate the NLRP3 inflammasome complex, leading to the production of interleukin-1β and an inflammatory response [138]. A specific phospholipid of mitochondrial inner membranes, cardiolipin, may play a role in activating the NLRP3 inflammasome outside mitochondria [139]. N-formylated methionine is the obligatory participant in the initiation of mitochondrial protein translation, since mitochondrial translation initiation factor 2 can only use this form of the protein. When an N-formyl peptide enters the cytosol or intercellular space, it binds to formyl peptide receptors, which, in turn, induce an inflammatory response [140]. It was shown that mitochondrial permeability transition and mitochondrial outer membrane VDAC-dependent permeabilization are involved in the release of mtDNA and other mtDAMPs [141,142]. 

It is important to consider the question of how mitochondria or mitochondrial components may ultimately be released from neurons. A recent study reported that mitochondria are indeed normally released from neurons at axonal terminals. These released mitochondria are then degraded by the surrounding glial cells [143]. Other previously recognized modes of mitochondria or mitochondrial components release outside cells include cell death events that proceed via necrosis or necroptosis pathways [131]. 

The problems of mitochondrial involvement, the disruption of their functions in the development of chronic systemic inflammation, and their role as an inducer of inflammation, while at the same time as a target of pathological inflammatory reactions, are of significant interest both in further research and in the development of mitochondria-directed therapy for various diseases, in particular T2D and Alzheimer’s disease.

## 7. Drugs for the Treatment of AD and T2D

Recently, in the development of potential drugs for the treatment of AD and T2D as diseases with multifactorial pathogenesis, the concept of a “magic shotgun”, that is, the model of “one disease-multiple targets”, is increasingly used. The simultaneous modulation of multiple targets using a well-coordinated pharmacological approach is essential to achieve the desired therapeutic effect [144,145]. In this case, the choice of a complex of targets and the determination of the design of screening studies are of great importance. The choice of targets may be based on the need to provide therapeutic effects to compensate for impaired functions and to eliminate characteristic disease-specific signs; in contrast, during the early stages of a disease, an important task is to eliminate the factors that are associated with an increased probability of developing the disease. Given the above, a study of the pathogenesis and specific characteristics of comorbid diseases and diseases that mutually provide an increase in the probability of pathology is of particular importance. The currently available treatments for AD and T2D have insufficient efficacy and are mainly compensatory and symptomatic by nature. Drugs that have a complex effect, combining a neuroprotective potential and an action on specific promising targets of either diabetes or AD, may be effective regarding other diseases. Thus, not only a targeted therapy of the underlying disease can be achieved but also the prevention and treatment of concomitant, related pathologies. Mitochondria are a very promising target for creating multi-target drugs to treat both diseases [146].

For AD, four drugs with a compensatory type of action are approved and actively used—three of them (galanthamine, rivastigmine, and donepezil) are acetylcholinesterase (AChE) inhibitors, while memantine is a low-affinity, non-competitive antagonist of N-methyl-D-aspartate (NMDA) type of ionotropic glutamate receptors [147]. The immunomodulatory/anti-inflammatory function of the cholinergic pathway plays a role in mild systemic inflammation, which is one of the pathogenetic factors in the development of both T2D and Alzheimer’s disease [148]. On the other hand, patients with AD and T2D have elevated plasma levels of acetylcholinesterase and butyrylcholinesterase [149], which are the markers of mild systemic inflammation [150]. The neuroprotective effect of donepezil, galantamine, and rivastigmine is associated with the stimulation of nicotinic acetylcholine receptor subtypes α7 and α4 (nAChRs). Moreover, the obtained data allow for the proposal that mitochondrial membranes, like neuronal membranes, contain at least the components of the receptor-related ion-permeable channels and some receptors, in particular nicotinic acetylcholine receptors α7, α9, and α10, which were shown to be able to regulate the activity of the VDAC and mPTP [148]. Therefore, it is not surprising that cholinesterase inhibitors, which were demonstrated to be effective anti-Alzheimer’s drugs, were also shown to be effective in the treatment of T2D [151,152].

A number of studies showed the effectiveness of some neuroprotective drugs in protecting pancreatic β-cells. A recently published review provided data confirming the antidiabetic and cytoprotective effects on the β-cells of a number of the neuroprotective drugs used: the low-molecular-weight NGF mimetic drug GK-2, the selective anxiolytic afobazole, and the antidepressants lithium chloride and lithium carbonate in the STZ model of T2D in rats [153]. mPTP inhibitors are obviously promising not only as neuroprotective agents but also as protectors against pancreatic β-cell death.

Given that in AD, which is considered as “type 3 diabetes”, there are common features with T2D, in particular regarding insulin signaling and the involvement of insulin in the regulation of amyloid plaques and neurofibrillary tangles formation, as well as the fact that T2D significantly increases the probability of developing AD; thus, it can be assumed that drugs effective for the treatment of T2D may be useful for the treatment of early forms of AD. Anti-diabetic drugs can be divided into two groups: (1) hypoglycemic agents, including insulin, sulfonylurea derivatives, and glinides, and (2) antihyperglycemic agents, including metformin, thiazolidinediones, dipeptidyl peptidase (DPP-4) inhibitors, glucagon-like peptide-1 (GLP-1) analogs, GLP-1 receptor agonists, and sodium-glucose transport protein 2 (SGLT-2) inhibitors.

Metformin was designated as a “baseline antiglycemic therapy” in patients with T2D who fail to achieve their target glycemic index despite diet and other lifestyle interventions. The main mechanisms of the antiglycemic action of this apparently multi-target drug are supposed to be AMPK activation, the stimulation of peripheral glucose uptake, and the blockade of key gluconeogenesis enzymes such as fructose-1,6-bisphosphatase-1 and glycerol-3-phosphate dehydrogenase [154]. A positive therapeutic effect of metformin on behavioral and emotional disorders in Alzheimer’s disease was also reported [155,156]. The potential mechanisms underlying the effectiveness of metformin for dementia prevention involve its antioxidant and anti-inflammatory effects [157,158]. However, possible negative effects of metformin were also described, i.e., the risk of developing cognitive impairment [159]. One of the mechanisms may be the metformin-induced stimulation of the expression of the APP and its processing with the accumulation of toxic forms of βA [160]. This effect can be prevented by the administration of insulin and the antioxidant curcumin.

DPP-4 inhibitors showed the ability not only to reduce glucose levels but also to prevent amyloid aggregates’ formation in patients with a combined diagnosis of T2D and AD [161]. Another study revealed that DPP-4 inhibitors decrease the rate of cognitive functions’ decline in patients [162].

Incretin/glucagon-like peptide-1 receptor agonists, developed and used for the treatment of T2D, can protect against dementia and Alzheimer’s disease, as shown in preclinical trials on AD models of cerebral amyloidosis APP/PS1, 3xTg-AD, and 5xFAD [133]. For the drugs liraglutide and exendin-4, a decrease in memory impairment and microglia-dependent neuroinflammation, a decrease in the accumulation of toxic forms of βA and in oxidative stress, the normalization of protein kinase A signaling and mitochondrial functions, and the activation of aerobic glycolysis in astrocytes, which contributes to neuronal survival, were shown [163].

One of the therapeutic strategies for the treatment of both AD and T2D is the identification of molecular targets associated with inflammation processes, including the use of already known drugs and strategies. Thus, a recent study using T2D mice on a high-fat-diet model demonstrated that transcranial exposure to near infrared light leads to a decrease in the levels of pro-inflammatory cytokines, a decrease in microglial activation, and an increase in the level of BDNF in animals’ brains [164].

In vivo experiments showed that the introduction of fibroblast growth factor 21 (FGF21) suppressed the aggregation of the tau protein and βA(1-42) in the mouse brain; significantly reduced the expression of Iba1, NF-κB, IL6, and IL8; and increased antioxidant enzymes in aging and diabetic mice. An in vitro experiment on SH-SY5Y neuroblastoma cells showed that FGF21 reduced aggregation by inhibiting NF-κB expression and stimulating AKT and AMPKα phosphorylation [165].

Using an experimental diabetes model of APPswe/PS1dE9 (APP/PS1) transgenic mice (reproducing the familial form of AD) on a high-fat diet, it was shown that the ad libitum administration of the non-steroidal anti-inflammatory drug dexibuprofen for 3 months reduced the levels of the molecular markers of inflammation and led to the mitigation of the symptomatic manifestations of the disease [166].

Antidiabetic drugs and PPAR-γ agonists such as thiazolidinediones, pioglitazone, and rosiglitazone can substantially stimulate neuronal bioenergetics and improve the memory in mouse models of AD. It was shown in a small group of patients with medium cognitive impairment that rosiglitazone improves the cognitive functions, but extensive clinical trials did not reveal any statistically significant efficacy [146].

Consistent with the fact that increased oxidative stress is common for both diseases is the search for effective AD and T2D medicines among known antioxidants. There is evidence of the potential efficacy of the natural antioxidant quercetin, which has antioxidant activity, in both AD [167] and diabetes [168]. The mitochondria-targeted tetrapeptide, Szeto-Schiller-31 (SS-31, H-D-Arg-Dmt-Lys-Phe-NH2), has significant therapeutic potential. SS-31 accumulates in the inner mitochondrial membrane in close proximity to the sites of ROS generation, regardless of the mitochondrial potential, and has a radical-binding, antioxidant effect [169]. SS-31 was able to reduce the permeability of the mitochondrial membrane and the oxidation of the mitochondria-specific diphosphatidylglycerol lipid—cardiolipin [170]. SS-31 reduced the level of ROS and the intensity of lipid peroxidation, and also had other positive effects in mice with a high-fat-diet T2D model [171,172]. In a cellular model of AD, neuroblastoma N2a transfected with AβPP (pCAX-AβPP Swe/Ind), and SS-31 reduced the production of both βA(1-40) and βA(1-42) and normalized neurite outgrowth [173]. In in vivo experiments on mice with the model of accelerated aging SAMP8 (a model of the sporadic form of AD), the SS-31 peptide not only reduced the level of H2O2 but also prevented the accumulation of Aβ(1-42) in brain cells and normalized the content of mitochondrial proteins—cyclophilin D, mitochondrial fission (DLP1 and Fis1), and mitochondrial fusion (Mfn2) proteins [174]. All the above-mentioned information allows us to conclude that a promising strategy for the treatment of age-related diseases that exhibit a significant level of comorbidity, such as T2DM and AD, involves influencing both specific targets for each disease and targets common to both diseases. The solution to this problem may be the creation of multi-target drugs that act on such targets. Mitochondria and the processes associated with the normalization of their functions seem to be an extremely promising target for the creation of antidiabetic and anti-Alzheimer’s drugs.

## 8. Conclusions

The pathogenesis of sporadic age-dependent forms of neurodegenerative diseases, in particular Alzheimer’s disease, is closely related to the mechanisms of aging and a number of diseases associated with metabolic disorders. T2D significantly increases the risk of developing cognitive impairments and AD. Both diseases are associated with increased oxidative stress, systemic inflammation, the appearance of amyloid-like aggregations of specific proteins, insulin resistance, and mitochondrial dysfunctions associated with the impairment of the electron transport chain, mitochondrial metabolism, and the regulation of cell death. It is important to understand the mechanisms by which defects in insulin signaling and metabolic disturbances can lead to accelerated AD progression. This knowledge is extremely important for developing a strategy for the search for new multi-target drugs for the treatment of T2D with the potential to prevent cognitive impairments and Alzheimer’s-type neurodegeneration.

## Data Availability

Not applicable.
